# Biliopancreatic limb obstruction after laparoscopic Roux‐en‐Y gastric bypass: a rare and potentially fatal condition

**DOI:** 10.1002/ccr3.1170

**Published:** 2017-09-08

**Authors:** Shireesh Saurabh

**Affiliations:** ^1^ Mercy Hospital 540 East Jefferson Street, Suite 205 Iowa City Iowa 52245

**Keywords:** Biliopancreatic limb, internal hernia, laparoscopic Roux‐en‐Y gastric bypass, morbid obesity, small bowel obstruction

## Abstract

Biliopancreatic limb obstruction after laparoscopic Roux‐en‐Y gastric bypass is a challenging diagnosis as the symptoms are very nonspecific. CT scan is the optimal study for evaluation. Early diagnosis and treatment is essential in reducing the morbidity and mortality associated with this condition.

## Question

**Figure 1:**
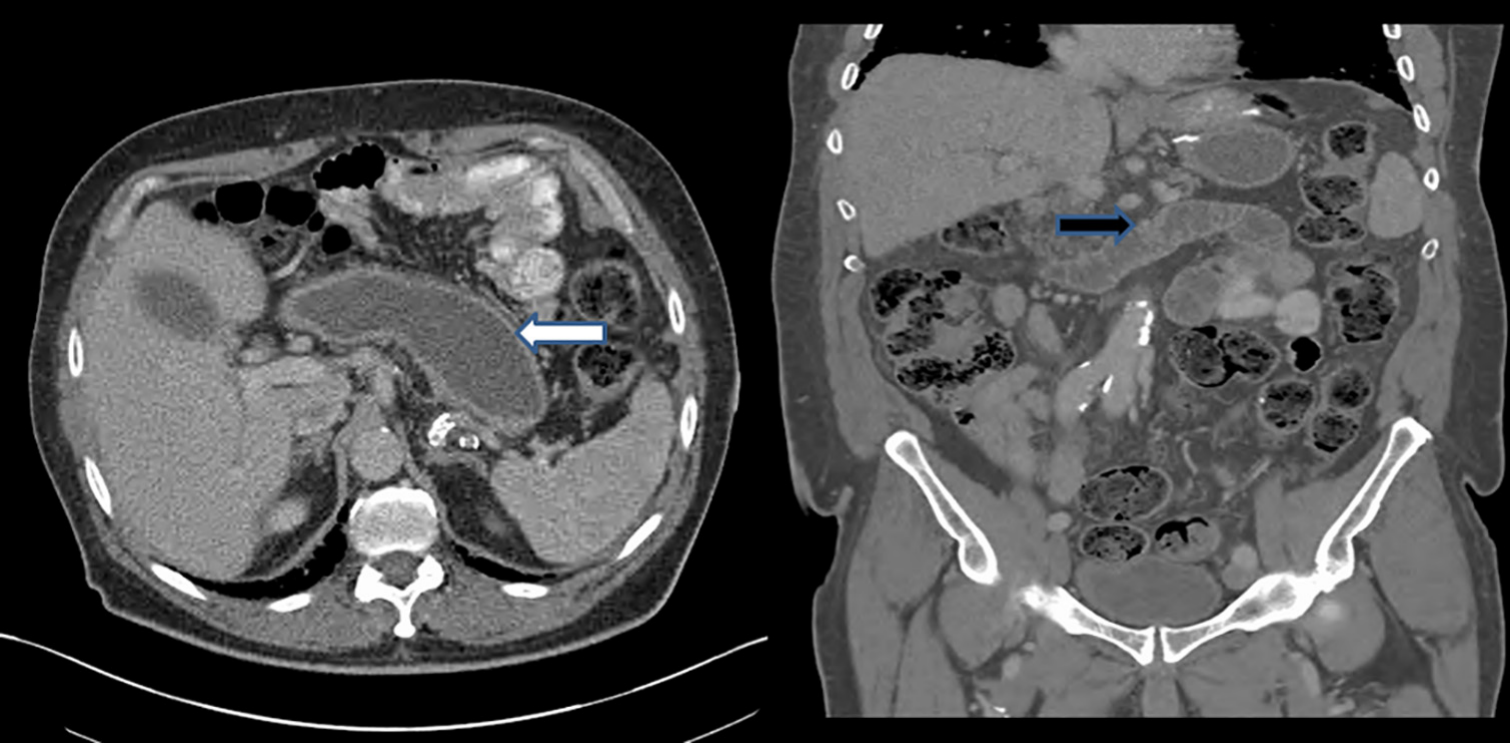
CT scan images show dilated gastric remnant (white arrow) and dilated biliopancreatic limb (black arrow).

**Figure 2:**
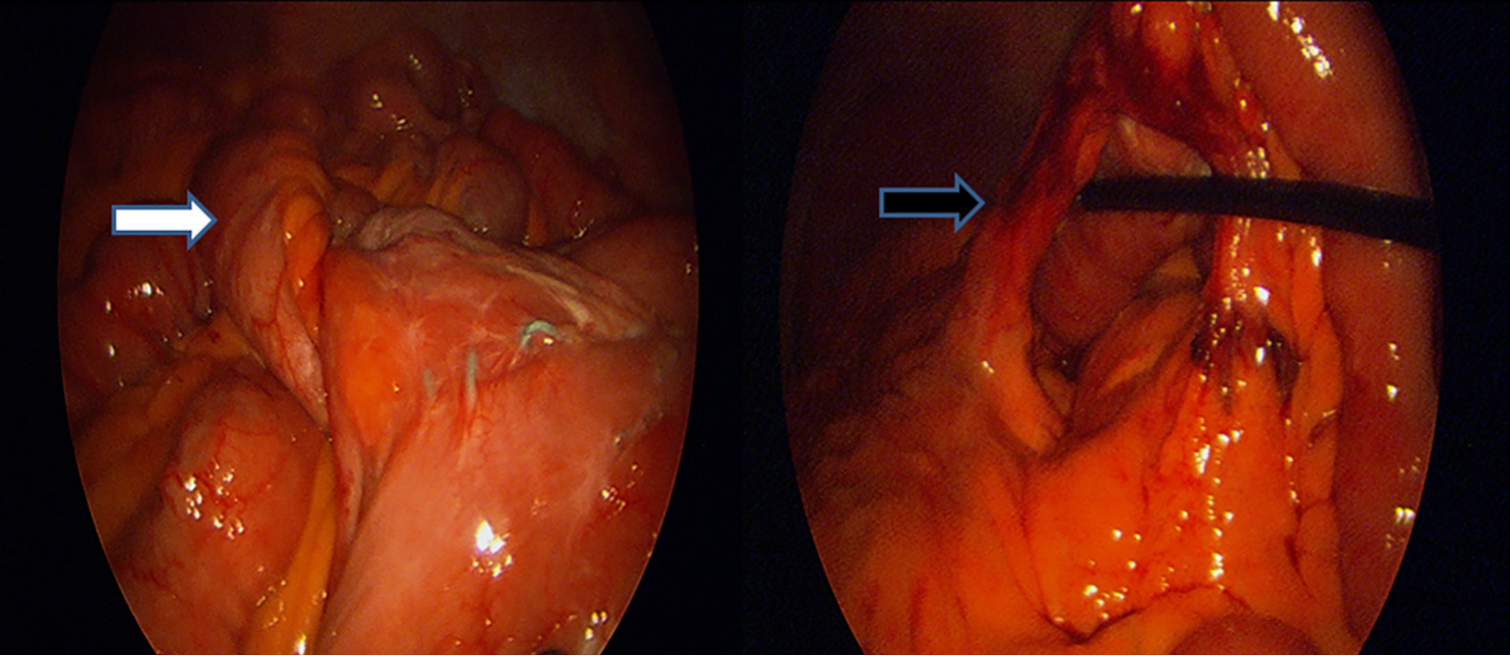
Intraoperative image demonstrates volvulus of biliopancreatic limb (white arrow) and large mesenteric defect (black arrow) at the level of Jejunojejunal anastomosis.

Sixty five‐year‐old male 18 month following Laparoscopic Roux‐en‐Y gastric bypass presented with abdominal pain and nausea. His vitals were stable, and on examination, he was tender in epigastric region. Computed tomography (CT) scan showed dilated biliopancreatic limb and remnant stomach (Fig. 1). There was also twisting of biliopancreatic limb at the jejunojejunal anastomosis site (Fig. 2). What is the etiology of this condition and how would you manage this patient?

## Answer

The etiology of the biliopancreatic limb obstruction includes adhesions, volvulus, stricture, and internal hernia at jejunojejunal anastomosis site 1, 2. Treatment includes emergent exploration either laparoscopically or open. The volvulus is reduced, adhesions are lysed, internal hernia is repaired, and bowel viability is assessed. If the bowel is ischemic, the patient will require bowel resection and occasionally revision of the jejunojejunal anastomosis.

## Authorship

SS: design, acquisition, analysis, and interpretation of the data, and drafting and the final approval of the work to be published.

## Conflict of Interest

None declared.
